# Quantitative Real-Time Polymerase Chain Reaction Measurement of *HLA-DRA* Gene Expression in Whole Blood Is Highly Reproducible and Shows Changes That Reflect Dynamic Shifts in Monocyte Surface HLA-DR Expression during the Course of Sepsis

**DOI:** 10.1371/journal.pone.0154690

**Published:** 2016-05-04

**Authors:** Sara Cajander, Elisabet Tina, Anders Bäckman, Anders Magnuson, Kristoffer Strålin, Bo Söderquist, Jan Källman

**Affiliations:** 1 Department of Infectious Diseases, Faculty of Medicine and Health, Örebro University, SE 70182 Örebro, Sweden; 2 Department of Clinical Research Laboratory, Faculty of Medicine and Health, Örebro University, Örebro, Sweden; 3 Clinical Epidemiology and Biostatistics, Faculty of Medicine and Health, Örebro University, Örebro, Sweden; 4 Department of Infectious Diseases, Karolinska University Hospital, Stockholm, Sweden; 5 Faculty of Medicine and Health, Örebro University, Örebro, Sweden; University of Pittsburgh, UNITED STATES

## Abstract

**Introduction:**

A decrease in the expression of monocyte surface protein HLA-DR (mHLA-DR), measured by flow cytometry (FCM), has been suggested as a marker of immunosuppression and negative outcome in severe sepsis. However, FCM is not always available due to sample preparation that limits its use to laboratory operational hours. In this prospective study we evaluated dynamic changes in mHLA-DR expression during sepsis in relation to changes in *HLA-DRA* gene expression and Class II transactivator *(CIITA)*, measured by quantitative Real-Time Polymerase Chain Reaction (qRT-PCR).

**Aims:**

The aims of this study were: 1. to validate the robustness of qRT-PCR measurement of *HLA-DRA-* and *CIITA*–mRNA expression, in terms of reproducibility; and 2. to see if changes in expression of these genes reflect changes in mHLA-DR expression during the course of severe and non-severe bacteraemic sepsis.

**Methods and Findings:**

Blood samples were collected from 60 patients with bacteraemic sepsis on up to five occasions during Days 1–28 after hospital admission. We found the reproducibility of the qRT-PCR method to be high by demonstrating low threshold variations (<0.11 standard deviation (SD)) of the qRT-PCR system, low intra-assay variation of Ct-values within triplicates (≤0.15 SD) and low inter-assay variations (12%) of the calculated target gene ratios. Our results also revealed dynamic *HLA-DRA* expression patterns during the course of sepsis that reflected those of mHLA-DR measured by FCM. Furthermore, *HLA-DRA* and mHLA-DR recovery slopes in patients with non-severe sepsis differed from those in patients with severe sepsis, shown by mixed model for repeated measurements (p<0.05). However, during the first seven days of sepsis, PCR-measurements showed a higher magnitude of difference between the two sepsis groups. Mean differences (95% CI) between severe sepsis (n = 20) and non-severe sepsis (n = 40) were; on day 1–2, *HLA-DRA* 0.40 (0.28–0.59) p<0.001, *CIITA* 0.48 (0.32–0.72) p = 0.005, mHLA-DR 0.63 (0.45–1.00) p = 0.04, day 7 *HLA-DRA* 0.59 (0.46–0.77) p<0.001, *CIITA* 0.56 (0.41–0.76) p<0.001, mHLA-DR 0.81 (0.66–1.00) p = 0.28.

**Conclusion:**

We conclude that qRT-PCR measurement of *HLA-DRA* expression is robust, and that this method appears to be preferable to FCM in identifying patients with severe sepsis that may benefit from immunostimulation.

## Introduction

Sepsis is a clinical syndrome arising from a systemic response following microbial infection [1], and represents a major healthcare problem due to high morbidity and mortality [2,3]. Until recently, the pathogenesis of severe sepsis with organ failure was thought to be collateral tissue damage caused by an exaggerated pro-inflammatory response to invading pathogens. Many clinical trials aiming to reduce mortality in sepsis by blocking pro-inflammatory activation, however, have failed to improve outcome, and this theory has been questioned [4]. In recent years, changes in the immune response during sepsis, with a shift towards an immunosuppressed hypo-inflammatory state, have been identified as important prognostic factors for unfavourable outcome [5–8].

Stimulation of a depressed immune response may thus provide us with a new weapon against secondary nosocomial infections [9–12]. However, immunostimulation during an inappropriate phase of sepsis could have deleterious consequences. It is important, therefore, that this form of sepsis treatment is based on biomarkers reflecting the underlying immune response. Expression of the surface protein HLA-DR on monocytes, from now on referred to as mHLA-DR, has been suggested as a global biomarker of clinically relevant immunosuppression in sepsis [13]. It is suitable as a marker of immunosuppression due to its important role in antigen presentation, representing a crucial immunological link between the innate and adaptive response. However, measurement of this marker by flow cytometry has practical disadvantages that complicate consecutive sampling in clinical studies. In particular, the sample must be prepared within 4 hours and analysed within 24 hours in order to assure accuracy [14,15]. In a previous paper we suggested that monitoring *HLA-DRA* gene expression, from now on referred to as *HLA-DRA*, could be a promising new approach since samples may be frozen pending analysis [14]. Our results showed that *HLA-DRA* measured by quantitative Real-Time PCR (qRT-PCR) correlated well with mHLA-DR in whole blood during Days 1–2 of sepsis. The transactivator of *HLA-DRA* gene transcription, a Class II transactivator (*CIITA*), was also found to be downregulated, and it is possible that *HLA-DRA* is under transcriptional control in sepsis [14].

Nevertheless, in order to be useful as biomarkers in sepsis, any method for measuring *HLA-DRA* and *CIITA*, expressed as a ratio to a reference gene, in this case peptidylpropylisomerase gene (PPIB), should be validated regarding its robustness during the course of sepsis. Specifically, the ability to generate reliable results with low inter- and intra-assay variation is essential if one is to compare patterns of gene expression.

The aims of this study were to investigate if qRT-PCR measurement of *HLA-DRA* and *CIITA* was robust in terms of reproducibility, and if the changes in gene expression reflected shifts in mHLA-DR during the course of sepsis.

## Materials and Methods

### Patient selection and sampling

This study was part of a larger single-centre study (“Dynamics of Sepsis”) conducted between 2011 and 2014 at Örebro University Hospital, Örebro, Sweden. Blood cultures were collected on admission (Day 0) from consecutive patients admitted to the Department of Infectious Diseases or Internal Medicine with suspected infection. Altogether 114 patients fulfilling criteria for sepsis and with blood cultures showing growth of pathogenic bacteria (bacteraemia) within Days 1–2 after admission were enrolled. Blood samples for mHLA-DR and *HLA-DRA*/*CIITA* measurement were taken at the same time on five occasions; 1–2, 3, 7±1, 14±2 and 28±4 days after admission.

Since the aim of the present study was to validate the robustness of the qRT-PCR method during the course of sepsis, we included patients from the larger study from whom blood cultures had been taken on at least three occasions. Most patients (n = 60), had samples taken Days 1–2, 7±1 and 14±2. If patients selected for inclusion also had samples taken Days 3 (n = 32) and 28±4 (n = 30), these data were also included in the analysis. The total number of blood samples taken for PCR measurements was 242. The definitions of sepsis severity in this study (sepsis, severe sepsis, and septic shock) were based on the criteria recommended by the American College of Chest Physicians/Society of Critical Care Medicine [16]. In the current study, evidence of bacterial infection was provided by positive blood cultures. All patients required fulfilment of two or more criteria of Systemic Inflammatory Response Syndrome (SIRS) according to the revised sepsis definitions in 2003 [17]. Severe sepsis was defined by evidence of hypoperfusion, organfailure or acute hypotension (systolic blood pressure ≤ 90mmHg). Septic shock was defined as persisting hypotension despite adequate fluid resuscitation in patients with severe sepsis. We defined “Non-severe sepsis” as a clinical condition where the criteria for sepsis were met, but not those for severe sepsis or septic shock. If criteria for severe sepsis were met, the acute change from baseline in Sequential [Sepsis-Related] Organ Failure Assessment Score (SOFA score) was calculated [18]. The baseline level was assumed to be zero in patients with no co-existing organ failure prior to the onset of sepsis.

### Sampling tubes

PAXgene Blood RNA tubes (Pre- AnalytiX GmbH, Qiagen group, Hilden, Germany) were used in the sampling of peripheral whole blood for PCR analysis. The PAXgene tubes were stored after sampling at -80°C pending further analysis. EDTA anticoagulant tubes were used in the sampling of peripheral whole blood for flow cytometry analysis of mHLA-DR. The samples for flow cytometry were immediately placed on ice and prepared within 4 hours.

### Control group

Blood samples from healthy blood donors at Örebro University Hospital were randomly collected and used as controls. For the qRT-PCR measurements we used 30 samples from a previous study [14]. In this control group, 73% were male (n = 22) and the median age was 49 years. For the mHLA-DR controls we used 61 samples, of which 30 hade been used in our previous study[14]. The sex distribution in this control group was 75% male (n = 46) and median age was 50 years.

### RNA isolation and cDNA preparation prior to qPCR

RNA was isolated from blood samples kept frozen 4–12 months after sampling in the majority of cases. In up to 30% of cases blood was kept frozen between two and three years. cDNA was prepared just prior to running the assays. The methods of RNA isolation and cDNA preparation have been described in detail in a previous paper by our group [14].

### Gene expression assays

The levels of expression of mRNA encoding a non-polymorphic region of the alpha-chain of the HLA-DR molecule, *HLA-DRA*, and mRNA coding for *CIITA*, [19], were obtained using qRT-PCR.

The cDNA (2 μL) was tested in the following TaqMan gene expression assays (FAM-labelled MGB probes) (Applied Biosystems/Life Technologies Europe BV, Stockholm, Sweden): HLADRA-A (Lot Hs00219575_ml), RefSeq: NM-019111.4, Amplicon 97 base pairs (bp); CIITA (Lot Hs00172094_ml), RefSeq: NM_000246.3, Amplicon 57 bp; and peptidylpropylisomerase B (PPIB) (Lot Hs00168719_ ml), RefSeq: NM_000942.4, Amplicon 67 bp.

The assays were run in triplicates (20uL) in a 96-well fast format on an ABI7900HT (Applied Biosystems (ABI)) real-time PCR machine for 40 cycles and analysed (RQ manager 1.2[ABI] with automatic threshold and detector centric mode, using water as negative control/calibrator) as in our previous publication [14]. In the event of error in a triplicate the whole dynamic series was rerun for all assays. Data were generated from 33 separately analysed plates using (SDS2.3 and RQ manager 1.2; Applied Biosystems).

For ratio calculations of the target genes (*HLA-DRA* and *CIITA)* in relation to the reference gene (PPIB), we calculated the ratio between target and reference genes using the expression; 2^-(ΔCt Target- ΔCtPPIB)^. This method was used since all three assays were equally efficient and thus directly comparable [14]. PPIB was used as the reference gene because of its previously described stability in inflammatory conditions [20].

### Flow cytometry

mHLA-DR was assessed at Days 1 or 2, 3, 7±1, 14±2 and 28±4 by standardised flow cytometry [15]. Antibody staining was performed within 4 hours after sampling using QuantiBRITETM Anti–HLA-DR PE*/Anti-Monocyte PerCP-Cy5.5 (BD Biosciences, San Jose, CA, USA) and QuantiBRITE^TM^ PE* (BD Biosciences) in accordance with the instructions of the manufacturer. An FC500 (Beckman Coulter, Fullerton, CA, USA) equipped with an argon laser (488 nm) and HeNe laser (633 nm) and EXPO 32 software. Kaluza v1.2, Beckman Coulter was used for data analysis, and results expressed as number of antibodies bound per cell (AB/c).

### Statistical analyses

The Shapiro-Wilk test was used to evaluate normal distribution and all analyses were performed after logarithmic transformation. Mixed models for repeated measurements were used to calculate the dynamic changes of mHLA-DR, *HLA-DRA* and *CIITA*, in relation to sepsis severity. The mixed model was used since there were values missing, with different numbers of patients participating at different sampling times. A heterogeneous first-order autoregressive correlation structure was chosen due to best model fit evaluated with Akaike information criteria (AIC). Independent variables were: Sepsis severity “severe sepsis/septic shock” (yes/no); time on a continuous scale; and the statistical interaction between time and severity. Based on the statistical interaction, we could evaluate whether the mean values of each marker revealed different gene/protein expressions over time, indicating different dynamic patterns. P<0.05 was regarded as statistically significant for the interaction test. If the interaction test was statistically significant, subsequent pairwise after-testing was applied at each sampling occasion. The p-values were Bonferroni corrected due to multiple comparisons. As the markers were evaluated on a log scale, geometric mean ratios between groups, with uncorrected 95% confidence intervals (CI) on original scale, are also presented, i.e. a mean ratio of 1 indicating no mean difference and a mean ratio of 2 indicating a mean level twice as high in one group compared to the other. Unpaired T-tests (Bonferroni corrected) were used for comparisons between septic patients and controls at each sampling occasion. Unpaired T-tests were used when assessing mean differences in mHLA-DR/HLA-DRA/CIITA expression in the groups categorized by high and low SOFA scores. All statistical analyses were performed with SPSS version 22 (IBM Corp., Armonk, NY, USA).

### Ethics

All patients included gave written informed consent, both to participate and for us to publish the findings. Ethical approval for the study was obtained from the Regional Ethics Review Board of Uppsala, Sweden.

## Results

### Demographic description of septic patients

The median age of the septic patients was 69 years and 28 (47%) were female. The Charlson comorbidity score [21] was > 1p in 36 patients (60%). Patients with severe sepsis and septic shock were grouped together (“severe sepsis/septic shock”) due to the small number of patients with septic shock, n = 2. In 20 of the 60 patients (33%) who were sampled on Days 1–2, 7±1 and 14±2, sepsis was classed as “severe sepsis/septic shock”. On sampling at Day 3, 11 of 32 patients (34%) and at Day 28, 11 of 30 patients (37%) had “severe sepsis/septic shock”. Ten of the 60 patients (17%) were admitted to the Intensive Care Unit. The SOFA scores on admission in the group of “severe sepsis/septic shock” ranged from 1–7 with a median value of 4.

### qRT-PCR method

When evaluating the reproducibility of the qRT-PCR method, the variation in cycle threshold (Ct) values and ratios were calculated in repeat runs of different samples (2–3 x, n = 38). Results showed an inter-assay variation in Ct values of 0.20 standard deviation (SD) for *HLA-DRA*, 0.16 SD for *CIITA* and 0.17 SD for *PPIB*. The coefficient of variation (CV) for ratio calculations was 12% for both *HLA-DRA* and *CIITA*.

The inter-assay variation of the reference gene was also evaluated in all samples (n = 242) and demonstrated a variation in Ct values of 0.62 SD with CV 2.4%.

The intra-assay mean variations of Ct-values within all assay triplicates for a sample was ≤0.15 SD, with an average of 0.04 SD.

The threshold setting of the qRT-PCR system was <0.11 SD for the analysed plates (n = 33) (HLA-DRA, 0.05 SD; CIITA 0.08 SD; PPIB 0.106 SD). Furthermore, negative controls (water plus assay-reaction) for all three assays and analysed qPCR-plates were negative (Ct = 40).

### Dynamic changes in mHLA-DR HLA-DRA and CIITA expression

Results of repeated analyses of mHLA-DR, HLA-DRA, and CIITA in individual patients are shown in S1–S3 Tables.

**Fig 1:**
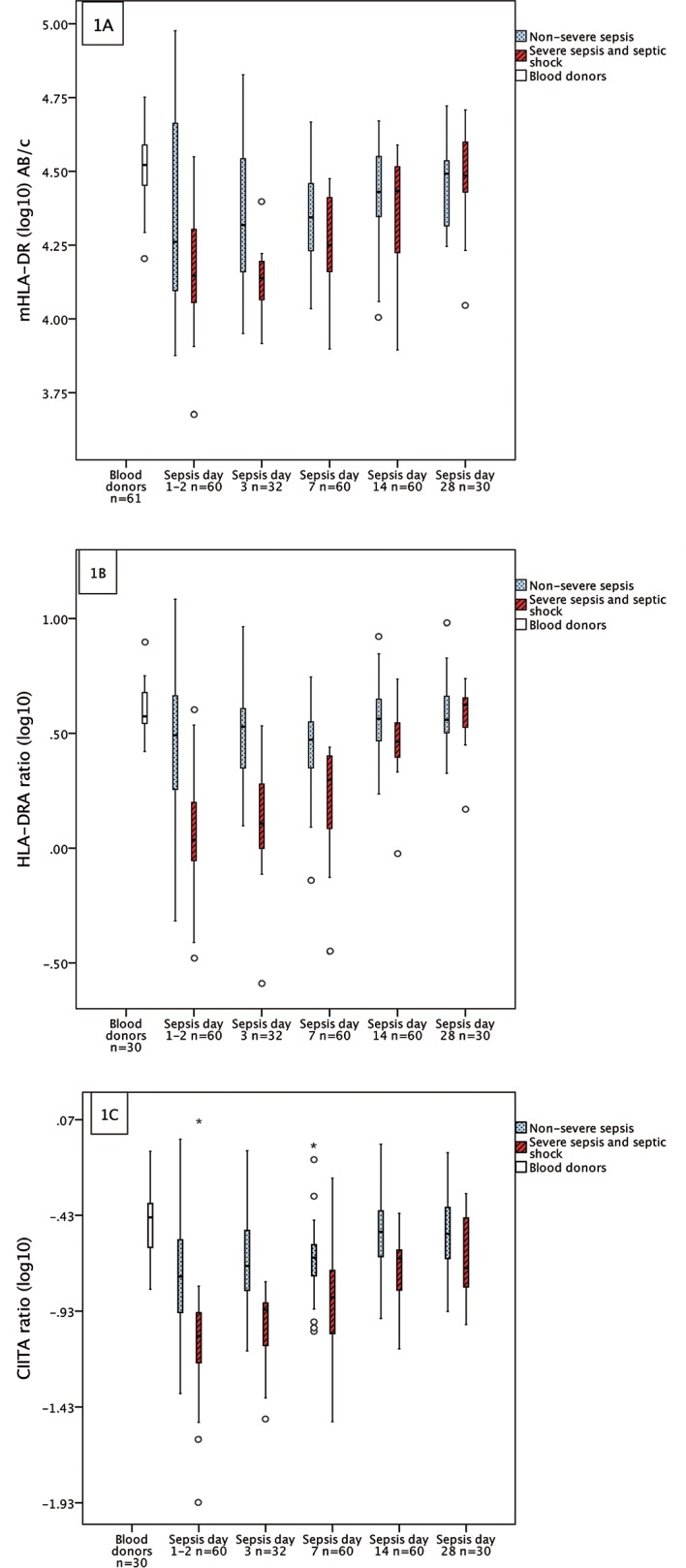
**(A) Monocyte HLA-DR (mHLA-DR), (B) HLA-DRA mRNA and (C) CIITA mRNA expression in bacteraemic sepsis categorized by sepsis severity on admission.** Interaction tests calculated by mixed model demonstrated significantly different linear associations over time between the severity groups in Fig 1A and 1B. Boxplots are defined by medians (line within the boxes), quartiles (box range), min-max (whiskers) if no outliers were present otherwise circle markers if outliers more than 1.5 box lengths from the box and asterisks (*) if outliers more than 3 box lengths from the box.

When evaluating the dynamics of expression using mixed models with all septic patients (n = 60), we found that mHLA-DR and *HLA-DRA* and *CIITA* all fell initially and subsequently increased significantly with time (p<0.001). The dynamic changes in expression over time for both mHLA-DR and *HLA-DRA*, showed significantly different patterns between the two sepsis severity groups (“non-severe” and”severe sepsis/septic shock”) (p = 0.036, p<0.001) as shown in Fig 1A and 1B. The expression over time for *CIITA* in the two severity groups had a similar pattern, however the interaction test was non-significant between sepsis severity and time (p = 0.060), Fig 1C.

The magnitude of difference between the sepsis severity groups was greater and the duration longer for *HLA-DRA* and *CIITA* compared to mHLA-DR, as shown in Table 1.

**Table 1:** The mean difference of mHLA-DR, *HLA-DRA* and *CIITA* between severity groups (severe sepsis and septic shock/non-severe sepsis), calculated on logarithmic scale, expressed as ratios and presented at each time point.

	Days	Mean differences (95% CI)	P-value unadjusted	P-value adjusted
**mHLA-DR**				
	1–2	**0.63 (0.45–1.00)**	**0.008**	**0.04**
	3	**0.58 (0.42–0.81)**	**0.002**	**0.01**
	7	0.81 (0.66–1.00)	0.06	0.28
	14	0.84 (0.68–1.04)	0.11	0.57
	28	0.52 (0.73–1.21)	0.62	1
***HLA-DRA***				
	1–2	**0.40 (0.28–0.59)**	**<0.001**	**<0.001**
	3	**0.44 (0.30–0.64)**	**<0.001**	**<0.001**
	7	**0.59 (0.46–0.77)**	**<0.001**	**<0.001**
	14	0.79 (0.64–0.97)	**0.026**	0.13
	28	1.00 (0.78–1.29)	0.988	1
***CIITA***				
	1–2	**0.48 (0.32–0.72)**	**0.001**	**0.005**
	3	**0.41 (0.27–0.62)**	**<0.001**	**<0.001**
	7	**0.56 (0.41–0.76)**	**<0.001**	**<0.001**
	14	**0.64 (0.51–0.81)**	**<0.001**	**<0.001**
	28	0.74 (0.52–1.03)	0.069	0.35

In pairwise testing of the differences between blood donors and septic patients at each time point, all biomarkers showed downregulated values until day 14 in the “severe sepsis/septic shock” group, although *HLA-DRA* and *CIITA* showed a greater magnitude of downregulation. The relative mean difference between patients with “severe sepsis/septic shock” and controls, at Days 1–2, was 0.45 for mHLA-DR, 0.30 for *HLA-DRA* and 0.24 for *CIITA*. Median values of blood donors and septic patients at each day of sampling are shown in Table 2.

**Table 2:** The median values and interquartile ranges (IQR) of mHLA-DR, *HLA-DRA* and *CIITA* at each sampling according to sepsis severity at hospital admission.

	mHLA-DR Median x10^(3)^ AB/c (IQR)		*HLA-DRA* Median ratio (IQR)		*CIITA* Median ratio (IQR)	
	Blood donors		Blood donors		Blood donors	
	33.2 (28.2–39.5) n = 61		3.75(3.49–4.76) n = 30		0.36 (0.25–0.43) n = 30	
Days after admission	Severe sepsis/septic shock	Non-Severe sepsis	Severe sepsis/septic shock	Non-Severe sepsis	Severe sepsis/septic shock	Non-Severe sepsis
**1–2**	13.9 (11.4–20.2) n = 20	18.2 (12.5–46.1) n = 40	1.09 (0.88–1.58) n = 20	3.10 (1.81–4.61) n = 40	0.09 (0.06–0.12) n = 20	0.18 (0.12–0.28) n = 40
**3**	13.7 (10.8–15.7) n = 11	20.8 (14.4–34.9) n = 21	1.28 (0.87–1.93) n = 11	3.38 (2.23–4.05) n = 21	0.12 (0.06–0.13) n = 11	0.20 (0.15–0.31) n = 21
**7**	17.8 (14.5–25.8) n = 20	22.1 (17.0–28.8) n = 40	1.98 (1.22–2.52) n = 20	2.97 (2.24–3.55) n = 40	0.14 (0.09–0.19) n = 20	0.22 (0.18–0.26) n = 40
**14**	27.2 (16.8–32.8) n = 20	26.9 (22.2–35.5) n = 40	2.91 (2.49–3.54) n = 20	3.66 (2.94–4.45) n = 40	0.22 (0.15–0.24) n = 20	0.30 (0.23–0.39) n = 40
**28**	30.6 (26.0–39.9) n = 11	31.0 (20.6–34.5) n = 19	4.21(2.98–4.55) n = 11	3.62 (3.04–4.62) n = 19	0.20 (0.15–0.38) n = 11	0.30 (0.21–0.45) n = 19

### HLA-DR expression in relation to SOFA score

In patients with severe sepsis/septic shock, the sepsis related organ failure was defined by the acute change in SOFA score on admission. In 19 of 20 patients with severe sepsis/septic shock, the baseline SOFA score was zero due to no pre-existing organ failure. One patient had an elevated baseline SOFA score of 2 due to renal insufficiency with habitual creatinine levels between 110–170 μmol/L prior to onset of sepsis. In this case the total SOFA score on admission, including baseline score, was 5. Since the acute change in SOFA score was 3, this patient was classified as having a SOFA score of 3 in our study.

**Fig 2:**
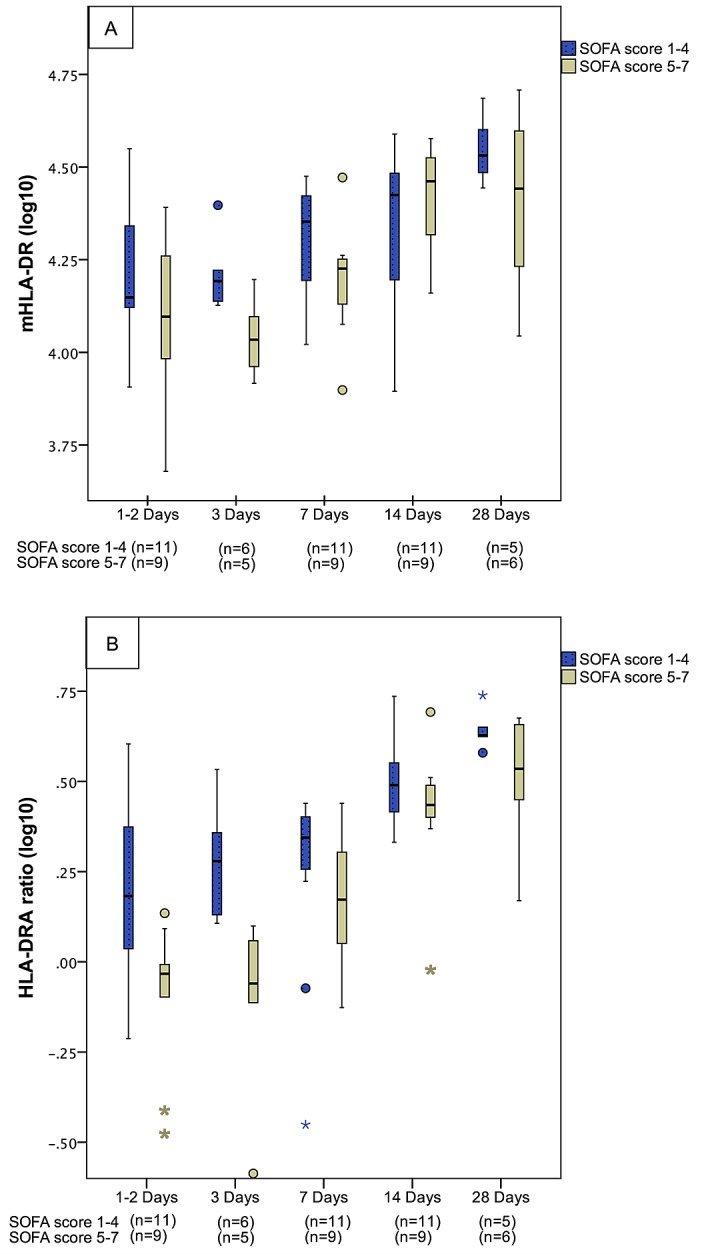
**Monocyte HLA-DR (A) and HLA-DRA mRNA expression (B) in 20 patients with bacteraemic severe sepsis categorized by the Sequential [Sepsis-Related] Organ Failure Assessment Score on admission.** On assessment 1–2 days after admission, the median levels of mHLA-DR (AB/c) and HLA-DRA (ratio) in patients with SOFA scores of ≥ 5 and < 5 were; 12400/14100 (p = 0.167) and 0.93/1.52 (p = 0.009) respectively. On day 3, median levels of mHLA-DR (AB/c) were 10800/15600 (p = 0.024) and HLA-DRA (ratio) 0.87/1.9 (p = 0.014) in the two SOFA score groups. No significant differences between groups were demonstrated on day 7, 14 or 28.

The 20 patients with severe sepsis/septic shock were stratified in two equally sized groups divided by high ≥ 5 (n = 9) or low <5 (n = 11) SOFA score. mHLA-DR and HLA-DRA demonstrated lower values in patients with SOFA score ≥ 5 when compared to SOFA score <5, as shown in Fig 2A and 2B.

CIITA expression did not differ significantly in the two SOFA score groups. Results of mHLA-DR, HLA-DRA, and CIITA related to SOFA score in individual patients are shown in S1–S3 Tables.

### Complete blood cell count in severe and non-severe sepsis

Levels of leukocytes, neutrophils and monocytes during the course of sepsis are shown in Table 3.

**Table 3:** Levels of leukocytes, neutrophils and monocytes during the course of sepsis.

	Leukocyte total cell count x10^(9)^/L Median (IQR)			Neutrophil cell count x10^(9)^/L Median (IQR)			Monocyte cell count x10^(9)^/L Median (IQR)	
Days after admission	Severe sepsis/Septic shock	Non-Severe sepsis		Severe sepsis/Septic shock	Non-Severe sepsis		Severe sepsis/Septic shock	Non-Severe sepsis
**1–2**	16.6 (9.8–18.5) n = 20	10.0 (6.4–14.5) n = 40		16.6 (9.8–18.5) n = 20	7.6 (4.7–11.8) n = 40	*	0.8 (0.40–1.2) n = 20	0.8 (0.5–1.2) n = 40
**3**	14.5 (8.0–25.0) n = 11	7.2 (5.4–10.4) n = 21	*	11.0 (6.4–19.3) n = 10	4.4 (3.5–7.6) n = 21	*	0.7 (0.5–1.4) n = 11	0.9 (0.5–1.0) n = 21
**7**	13.2 (11.8–19.1) n = 19	9.0 (6.3–11.5) n = 38	*	10.0 (8.3–12.0) n = 18	6.2 (4.0–8.1) n = 37	*	1.0 (0.8–1.3) n = 19	0.8 (0.5–1.0) n = 38
**14**	9.7 (7.1–11.8) n = 19	7.6 (6.3–9.8) n = 39		7.5 (4.8–9.6) n = 19	4.7 (3.6–6.3) n = 38	*	0.8 (0.6–1.0) n = 19	0.6 (0.5–0.8) n = 39
**28**	8.8 (5.8–13.1) n = 11	6.9 (5.1–8.1) n = 19		4.6 (3.1–9.4) n = 11	4.0 (3.0–5.3) n = 19		0.7 (0.6–0.9) n = 11	0.6 (0.5–0.8) n = 19

* Significant mean difference between severe sepsis/septic shock and non-severe sepsis (p<0.05, Bonferroni adjusted).

## Discussion

This study demonstrates that monitoring *HLA-DRA* and *CIITA* gene expression by qRT-PCR is a robust method in terms of reproducibility and repeatability as shown by low inter- and intra-assay variations and stable threshold settings. Moreover, a prerequisite of being able to achieve reliable PCR results when measuring the rapid alterations over time in sepsis is also that the reference gene is stable. Our results demonstrated that PPIB, during the course of bacteraemic sepsis, was as stable as the blood donor controls without sepsis. This supports previous data demonstrating stability of PPIB in inflammatory conditions [20] further supporting PPIB as a suitable reference gene for mRNA quantification in peripheral whole blood from septic patients. According to our results, dynamic variations in *HLA-DRA* and *CIITA* gene expression can be reliably detected during the course of sepsis. Nevertheless, since the coefficient of ratio variation is 12%, changes in expression should be greater than this when interpreting significant individual differences over time.

In order to evaluate the robustness of the qRT-PCR method, when comparing dynamic changes of *HLA-DRA* and *CIITA* with the gold standard mHLA-DR, we applied interaction tests over time. Based on the statistical interaction tests, we evaluated whether or not the means of mHLA-DR, and *HLA-DRA* showed different recovery patterns over time and whether patterns differed between”non-severe sepsis” and “severe sepsis/septic shock”. Our results showed that the dynamic changes in *HLA-DRA* were similar to those of mHLA-DR measured by FCM when testing for both time and severity (Fig 1A and 1B). This implies that highly dynamic changes in gene expression in sepsis over time are detectable at the mRNA level (*HLA-DRA*) as well as in the surface expression of mHLA-DR.

Interestingly, some differences regarding gene dynamics were seen. *HLA-DRA* was more indicative of initial sepsis severity. In particular, mHLA-DR discriminated between severe and non-severe sepsis in the early stage of sepsis only, while *HLA-DRA-* and *CIITA*-mRNA levels measured by qRT-PCR, showed prolonged divergence between our severity groups. As shown in Fig 1, *HLA-DRA* and *CIITA* decreased to a greater extent in the “severe sepsis/septic shock” group and to a lesser extent in the “non-severe sepsis” group, than did mHLA-DR measured by flow cytometry. In the PCR-based measurements, significant differences between the two severity groups were seen up to 14 days after admission. This was in contrast to the FCM-based measurements of mHLA-DR that only discriminated between severity groups up to Day 3. Moreover, HLA-DRA measured by qRT-PCR discriminated better between high and low SOFA scores among the patients with severe sepsis and septic shock, than did mHLA-DR measured by flow cytometry. This feature of *HLA-DRA* could make PCR assessment a favourable biomarker method to distinguish between severe and non-severe disease during the course of sepsis. Whether or not this difference has prognostic implications should be the subject of future studies. Furthermore, to our knowledge, the dynamics of HLA-DR gene expression in relation to monocyte surface expression of HLA-DR has not been studied before. However, the results we present regarding disease severity are supported in the recently published genome-wide transcriptomic study on dynamic changes in septic patients showing that sepsis severity affects the magnitude and duration of acute inflammatory changes [22].

When monitoring gene expression in whole blood we measured overall HLA-DR expression in all immune cells, not only monocytes, which might have contributed to the greater discrimination between our severity groups using the PCR-guided technique. However, the dynamics in monocyte cell count does not seem to explain these differences since the monocyte levels were quite stable during the course of sepsis and did not differ between the two severity groups (Table 3). The proportion of HLA-DR up regulation in specific immune cells might have differed between patients with severe or non-severe sepsis, but we have not measured these effects in the current study.

Randomised controlled multicentre trials evaluating immunostimulation in sepsis, guided by expression of the dominating immunophenotype, are warranted but difficult to conduct. Gouel-Cheron et al recently published a study describing how daily measurement of mHLA-DR predicts forthcoming sepsis in trauma patients by monitoring dynamic changes between Days 2 and 3 [23]. However, the authors highlighted difficulties in recruiting study patients due to the limitations imposed by flow cytometry, i.e. not allowing sampling outside laboratory operational hours. This prolonged the time period of the study.

In this study, we have shown that *HLA-DRA* gene expression in whole blood, measured by qRT-PCR, can be used as a surrogate for the gold standard mHLA-DR, despite samples being kept frozen at -80°C pending analysis. This approach allows sampling around the clock, and samples may also be analysed at collaborating laboratories. Based on this, we were able to demonstrate down-regulation of *HLA-DRA* and other HLA-DR-related gene products, consistent with the sepsis study by Cazalis et al demonstrating that HLA-DR mRNA markers act as independent predictors of mortality when measured Day 3 [24]. Moreover, individual dynamic changes in expression of these biomarkers between two points in time may be of even greater value, enabling us to study the heterogeneity of immune responses in septic patients. In this study we have shown that monitoring of *HLA-DRA* expression in whole blood using qRT-PCR is reliable for the detection of rapid dynamic changes in expression during bacteraemic sepsis. However, a limitation to this study is that blood donors were used as controls when assessing the time to normalisation of the studied HLA-DR markers. Since the blood donor controls had a different age and sex distribution compared to septic patients, firm conclusions cannot be drawn regarding these differences. Nevertheless, age or sex was not clearly associated with levels of mHLA-DR-, *HLA-DRA-* or *CIITA*-expression in the controls of the current study (data not shown).

In summary, we have shown that monitoring of *HLA-DRA* at gene expression level using qRT-PCR was robust and showed dynamic changes over time in sepsis similar to the current gold standard of mHLA-DR monitoring by flow cytometry, but with a lesser degree of overlapping between severity groups. Consequently, we conclude that monitoring *HLA-DRA* by qRT-PCR allows detection of dynamic changes in the immune state in sepsis, and this technique may be used in future studies aiming to identify high-risk patients who might benefit from immunostimulation therapy.

## Supporting Information

S1 Table HLA-DR antibodies bound per monocyte cell (mHLA-DR) in bacteraemic infection categorized by initial sepsis severity. (DOCX)

S2 Table HLA-DRA mRNA ratio (HLA-DRA/PPIB-reference) in bacteraemic infection categorized by initial sepsis severity. (DOCX)

S3 Table *CIITA* mRNA ratio (CIITA/PPIB-reference) expression in bacteraemic infection categorized by sepsis severity. (DOCX)

## References

[1] Angus DC, van der Poll T (2013) Severe sepsis and septic shock. N Engl J Med 369: 840–851. 10.1056/NEJMra1208623 23984731

[2] Iwashyna TJ, Ely EW, Smith DM, Langa KM (2010) Long-term cognitive impairment and functional disability among survivors of severe sepsis. JAMA 304: 1787–1794. 10.1001/jama.2010.1553 20978258 PMC3345288

[3] Angus DC, Linde-Zwirble WT, Lidicker J, Clermont G, Carcillo J, Pinsky MR (2001) Epidemiology of severe sepsis in the United States: analysis of incidence, outcome, and associated costs of care. Critical Care Medicine 29: 1303–1310. 11445675 10.1097/00003246-200107000-00002

[4] Hotchkiss RS, Monneret G, Payen D (2013) Sepsis-induced immunosuppression: from cellular dysfunctions to immunotherapy. Nat Rev Immunol 13: 862–874. 10.1038/nri3552 24232462 PMC4077177

[5] Boomer JS, To K, Chang KC, Takasu O, Osborne DF, Walton AH, et al (2011) Immunosuppression in patients who die of sepsis and multiple organ failure. JAMA 306: 2594–2605. 10.1001/jama.2011.1829 22187279 PMC3361243

[6] Walton AH, Muenzer JT, Rasche D, Boomer JS, Sato B, Brownstein BH, et al (2014) Reactivation of multiple viruses in patients with sepsis. PLoS One 9: e98819 10.1371/journal.pone.0098819 24919177 PMC4053360

[7] Landelle C, Lepape A, Voirin N, Tognet E, Venet F, Bohe J, et al (2010) Low monocyte human leukocyte antigen-DR is independently associated with nosocomial infections after septic shock. Intensive Care Med 36: 1859–1866. 10.1007/s00134-010-1962-x 20652682

[8] Kalil AC, Florescu DF (2009) Prevalence and mortality associated with cytomegalovirus infection in nonimmunosuppressed patients in the intensive care unit. Crit Care Med 37: 2350–2358. 10.1097/CCM.0b013e3181a3aa43 19531944

[9] Meisel C, Schefold JC, Pschowski R, Baumann T, Hetzger K, Gregor J, et al (2009) Granulocyte-macrophage colony-stimulating factor to reverse sepsis-associated immunosuppression: a double-blind, randomized, placebo-controlled multicenter trial. American Journal of Respiratory and Critical Care Medicine 180: 640–648. 10.1164/rccm.200903-0363OC 19590022

[10] Payen D, Monneret G, Hotchkiss R (2013) Immunotherapy—a potential new way forward in the treatment of sepsis. Crit Care 17: 118 10.1186/cc12490 23425441 PMC4056021

[11] Delsing CE, Gresnigt MS, Leentjens J, Preijers F, Frager FA, Kox M, et al (2014) Interferon-gamma as adjunctive immunotherapy for invasive fungal infections: a case series. BMC Infect Dis 14: 166 10.1186/1471-2334-14-166 24669841 PMC3987054

[12] Spies C, Luetz A, Lachmann G, Renius M, von Haefen C, Wernecke KD, et al (2015) Influence of Granulocyte-Macrophage Colony-Stimulating Factor or Influenza Vaccination on HLA-DR, Infection and Delirium Days in Immunosuppressed Surgical Patients: Double Blind, Randomised Controlled Trial. PLoS One 10: e0144003 10.1371/journal.pone.0144003 26641243 PMC4671639

[13] Monneret G, Venet F (2015) Sepsis-induced immune alterations monitoring by flow cytometry as a promising tool for individualized therapy. Cytometry B Clin Cytom. 10.1002/cyto.b.21270 26130241

[14] Cajander S, Backman A, Tina E, Stralin K, Soderquist B, Kallman J (2013) Preliminary results in quantitation of HLA-DRA by real-time PCR: a promising approach to identify immunosuppression in sepsis. Crit Care 17: R223 10.1186/cc13046 24093602 PMC4057202

[15] Docke WD, Hoflich C, Davis KA, Rottgers K, Meisel C, Kiefer P, et al (2005) Monitoring temporary immunodepression by flow cytometric measurement of monocytic HLA-DR expression: a multicenter standardized study. Clin Chem 51: 2341–2347. 16214828 10.1373/clinchem.2005.052639

[16] Bone RC, Balk RA, Cerra FB, Dellinger RP, Fein AM, Knaus WA, et al (1992) Definitions for sepsis and organ failure and guidelines for the use of innovative therapies in sepsis. The ACCP/SCCM Consensus Conference Committee. American College of Chest Physicians/Society of Critical Care Medicine. Chest 101: 1644–1655. 1303622 10.1378/chest.101.6.1644

[17] Levy MM, Fink MP, Marshall JC, Abraham E, Angus D, Cook D, et al (2003) 2001 SCCM/ESICM/ACCP/ATS/SIS International Sepsis Definitions Conference. Crit Care Med 31: 1250–1256. 12682500 10.1097/01.CCM.0000050454.01978.3B

[18] Vincent JL, Moreno R, Takala J, Willatts S, De Mendonca A, Bruining H, et al (1996) The SOFA (Sepsis-related Organ Failure Assessment) score to describe organ dysfunction/failure. On behalf of the Working Group on Sepsis-Related Problems of the European Society of Intensive Care Medicine. Intensive Care Med 22: 707–710. 8844239 10.1007/BF01709751

[19] Hanna S, Etzioni A (2014) MHC class I and II deficiencies. J Allergy Clin Immunol 134: 269–275. 10.1016/j.jaci.2014.06.001 25001848

[20] Pachot A, Blond JL, Mougin B, Miossec P (2004) Peptidylpropyl isomerase B (PPIB): a suitable reference gene for mRNA quantification in peripheral whole blood. J Biotechnol 114: 121–124. 15464605 10.1016/j.jbiotec.2004.07.001

[21] Charlson ME, Pompei P, Ales KL, MacKenzie CR (1987) A new method of classifying prognostic comorbidity in longitudinal studies: development and validation. J Chronic Dis 40: 373–383. 3558716 10.1016/0021-9681(87)90171-8

[22] Cazalis MA, Lepape A, Venet F, Frager F, Mougin B, Vallin H, et al (2014) Early and dynamic changes in gene expression in septic shock patients: a genome-wide approach. Intensive Care Med Exp 2: 20 10.1186/s40635-014-0020-3 26215705 PMC4512996

[23] Gouel-Cheron A, Allaouchiche B, Floccard B, Rimmele T, Monneret G (2015) Early daily mHLA-DR monitoring predicts forthcoming sepsis in severe trauma patients. Intensive Care Med. 10.1007/s00134-015-4045-1 26359166

[24] Cazalis MA, Friggeri A, Cave L, Demaret J, Barbalat V, Cerrato E, et al (2013) Decreased HLA-DR antigen-associated invariant chain (CD74) mRNA expression predicts mortality after septic shock. Crit Care 17: R287 10.1186/cc13150 24321376 PMC4056003

