# Supplementary material for: Quantitative Real-Time Polymerase Chain Reaction Measurement of HLA-DRA Gene Expression in Whole Blood Is Highly Reproducible and Shows Changes That Reflect Dynamic Shifts in Monocyte Surface HLA-DR Expression during the Course of Sepsis
Source: PLoS One. 2016 May 4;11(5):e0154690. doi: 10.1371/journal.pone.0154690 (PMC4856385; doi:10.1371/journal.pone.0154690)
Supplement: S1 Table — (DOCX) [file pone.0154690.s001.docx]

**S1 Table. HLA-DR antibodies bound per monocyte cell (mHLA-DR) in bacteraemic infection categorized by initial sepsis severity.**

| **ID** | **Severity** | **SOFA-**  **score** ^a^ | **mHLA-DR**  **Day 1-2** | **mHLA-DR**  **Day 3** ^b^ | **mHLA-DR**  **Day 7** | **mHLA-DR**  **Day 14** | **mHLA-DR**  **Day 28** ^b^ |
| --- | --- | --- | --- | --- | --- | --- | --- |
| 1 | Septic shock | 6 | 18 534 |  | 17 774 | 37 759 |  |
| 2 | Septic shock | 5 | 9 609 | 8 251 | 14 868 | 33 484 | 51 024 |
| 3 | Severe sepsis | 7 | 4 772 |  | 7 911 | 22 299 |  |
| 4 | Severe sepsis | 7 | 8 992 |  | 18 269 | 33 040 | 29 381 |
| 5 | Severe sepsis | 7 | 12 837 | 12 488 | 17 834 | 20 767 |  |
| 6 | Severe sepsis | 6 | 11 489 | 10 816 | 29 633 | 28 939 | 39 571 |
| 7 | Severe sepsis | 5 | 12 482 | 9 158 | 11 902 | 14 444 | 11 063 |
| 8 | Severe sepsis | 5 | 18 204 | 15 717 | 16 829 | 36 342 | 26 019 |
| 9 | Severe sepsis | 5 | 24 605 |  | 13 493 | 16 416 | 17 059 |
| 10 | Severe sepsis | 4 | 8 062 |  | 10 507 | 7 849 |  |
| 11 | Severe sepsis | 4 | 21 831 | 13 742 | 26 052 | 30 831 | 30 586 |
| 12 | Severe sepsis | 3 | 11 237 |  | 26 856 | 30 043 |  |
| 13 | Severe sepsis | 3 | 12 529 | 13 377 | 22 513 | 27 786 |  |
| 14 | Severe sepsis | 2 | 14 058 |  | 14 795 | 17 137 |  |
| 15 | Severe sepsis | 2 | 22 048 |  | 14 145 | 8 580 |  |
| 16 | Severe sepsis | 2 | 35 423 | 24 942 | 25 473 | 32 504 | 33 984 |
| 17 | Severe sepsis | 1 | 13 944 | 15 614 | 28 349 | 38 798 | 39 886 |
| 18 | Severe sepsis | 1 | 14 014 | 15 501 | 16 530 | 24 844 | 27 772 |
| 19 | Severe sepsis | 1 | 14 972 | 16 654 | 29 841 | 26 562 | 48 475 |
| 20 | Severe sepsis | 1 | 33 024 |  | 19 206 | 14 356 |  |
| 21 | Non-severe sepsis | | 7 515 | 9 193 | 26 143 | 24 809 | 28 650 |
| 22 | Non-severe sepsis | | 8 321 |  | 16 554 | 38 354 |  |
| 23 | Non-severe sepsis | | 9 710 |  | 20 211 | 22 132 |  |
| 24 | Non-severe sepsis | | 9 712 | 8 922 | 10 834 | 10 102 | 17 599 |
| 25 | Non-severe sepsis | | 10 518 |  | 11 802 | 11 451 |  |
| 26 | Non-severe sepsis | | 11 010 |  | 21 153 | 28 246 |  |
| 27 | Non-severe sepsis | | 11 058 |  | 27 155 | 26 467 |  |
| 28 | Non-severe sepsis | | 11 474 |  | 15 163 | 20 648 |  |
| 29 | Non-severe sepsis | | 11 708 | 12 538 | 14 510 | 18 678 | 35 339 |
| 30 | Non-severe sepsis | | 12 303 | 10 302 | 16 313 | 35 687 |  |
| 31 | Non-severe sepsis | | 12 649 |  | 16 347 | 17 504 |  |
| 32 | Non-severe sepsis | | 13 148 | 14 438 | 18 725 | 37 318 | 34 184 |
| 33 | Non-severe sepsis | | 13 661 |  | 12 812 | 21 069 |  |
| 34 | Non-severe sepsis | | 15 202 |  | 32 265 | 38 835 |  |
| 35 | Non-severe sepsis | | 15 327 |  | 31 824 | 32 710 |  |
| 36 | Non-severe sepsis | | 16 024 | 17 548 | 46 448 | 34 941 | 32 554 |
| 37 | Non-severe sepsis | | 16 610 |  | 17 509 | 21 503 |  |
| 38 | Non-severe sepsis | | 16 786 |  | 36 333 | 37 626 |  |
| 39 | Non-severe sepsis | | 16 973 | 19 238 | 23 566 | 22 825 | 25 495 |
| 40 | Non-severe sepsis | | 18 151 | 25 592 | 18 855 | 27 391 | 31 165 |
| 41 | Non-severe sepsis | | 18 335 | 16 303 | 23 393 | 24 941 | 19 498 |
| 42 | Non-severe sepsis | | 22 429 | 11 549 | 11 571 | 20 315 | 20 771 |
| 43 | Non-severe sepsis | | 22 912 |  | 18 372 | 25 191 |  |
| 44 | Non-severe sepsis | | 25 506 |  | 20 223 | 26 189 |  |
| 45 | Non-severe sepsis | | 26 111 | 20 797 | 34 307 | 35 338 | 34 486 |
| 46 | Non-severe sepsis | | 31 730 | 34 906 | 14 453 | 20 888 | 18 847 |
| 47 | Non-severe sepsis | | 32 044 | 32 971 | 21 093 | 46 817 | 32 452 |
| 48 | Non-severe sepsis | | 35 087 | 19 918 | 27 753 | 25 734 | 19 548 |
| 49 | Non-severe sepsis | | 41 822 |  | 27 499 | 41 334 |  |
| 50 | Non-severe sepsis | | 42 761 | 36 082 | 22 241 | 23 668 | 20 553 |
| 51 | Non-severe sepsis | | 49 501 |  | 26 076 | 31 709 |  |
| 52 | Non-severe sepsis | | 55 075 | 26 347 | 33 804 | 27 897 | 31 036 |
| 53 | Non-severe sepsis | | 55 921 |  | 29 795 | 45 021 |  |
| 54 | Non-severe sepsis | | 56 764 | 52 059 | 35 210 | 33 924 | 35 532 |
| 55 | Non-severe sepsis | | 57 473 |  | 18 243 | 22 325 |  |
| 56 | Non-severe sepsis | | 61 661 | 67 172 | 22 782 | 33 419 | 26 332 |
| 57 | Non-severe sepsis | | 66 962 | 45 012 | 30 875 | 37 658 | 36 124 |
| 58 | Non-severe sepsis | | 69 638 | 30 270 | 21 912 | 30 906 | 52 697 |
| 59 | Non-severe sepsis | | 84 113 |  | 45 160 | 43 671 |  |
| 60 | Non-severe sepsis | | 94 755 | 35 131 | 27 081 | 25 672 |  |

^a^ Determined in cases with severe sepsis/septic shock.

^b^ Empty fields indicate missing data and no performed analysis.
