# Supplementary material for: Quantitative Real-Time Polymerase Chain Reaction Measurement of HLA-DRA Gene Expression in Whole Blood Is Highly Reproducible and Shows Changes That Reflect Dynamic Shifts in Monocyte Surface HLA-DR Expression during the Course of Sepsis
Source: PLoS One. 2016 May 4;11(5):e0154690. doi: 10.1371/journal.pone.0154690 (PMC4856385; doi:10.1371/journal.pone.0154690)
Supplement: S2 Table — (DOCX) [file pone.0154690.s002.docx]

**S2 Table. HLA-DRA mRNA ratio (HLA-DRA/PPIB-reference) in bacteraemic infection categorized by initial sepsis severity.**

| **ID** | **Severity** | **SOFA-**  **score** ^a^ | **HLA-DRA**  **Day 1-2** | **HLA-DRA**  **Day 3** ^b^ | **HLA-DRA**  **Day 7** | **HLA-DRA**  **Day 14** | **HLA-DRA**  **Day 28** ^b^ |
| --- | --- | --- | --- | --- | --- | --- | --- |
| 1 | Septic shock | 6 | .84 |  | 1.32 | 4.92 |  |
| 2 | Septic shock | 5 | .33 | .26 | .75 | 2.76 | 3.95 |
| 3 | Severe sepsis | 7 | .80 |  | 1.59 | 2.52 |  |
| 4 | Severe sepsis | 7 | .39 |  | 2.53 | 3.24 | 4.74 |
| 5 | Severe sepsis | 7 | 1.24 | 1.14 | 2.01 | 2.72 |  |
| 6 | Severe sepsis | 6 | .98 | .87 | 2.75 | 2.34 | 4.55 |
| 7 | Severe sepsis | 5 | .93 | .77 | 1.12 | 2.52 | 1.48 |
| 8 | Severe sepsis | 5 | .98 | 1.26 | 1.49 | 3.08 | 2.98 |
| 9 | Severe sepsis | 5 | 1.36 |  | 1.06 | .95 | 2.81 |
| 10 | Severe sepsis | 4 | .61 |  | 2.28 | 3.54 |  |
| 11 | Severe sepsis | 4 | 2.44 | 1.87 | 2.21 | 2.46 | 4.25 |
| 12 | Severe sepsis | 3 | .95 |  | .35 | 3.06 |  |
| 13 | Severe sepsis | 3 | 1.48 | 2.28 | .84 | 2.75 |  |
| 14 | Severe sepsis | 2 | 1.52 |  | 1.67 | 3.50 |  |
| 15 | Severe sepsis | 2 | 4.02 |  | 1.95 | 2.15 |  |
| 16 | Severe sepsis | 2 | 2.30 | 3.41 | 2.75 | 5.45 | 5.48 |
| 17 | Severe sepsis | 1 | 1.04 | 1.28 | 2.18 | 2.34 | 3.80 |
| 18 | Severe sepsis | 1 | 1.14 | 1.35 | 2.67 | 3.59 | 4.48 |
| 19 | Severe sepsis | 1 | 1.65 | 1.93 | 2.51 | 5.06 | 4.21 |
| 20 | Severe sepsis | 1 | 3.43 |  | 2.53 | 3.09 |  |
| 21 | Non-severe sepsis | | 1.54 | 1.67 | 2.85 | 2.30 | 3.58 |
| 22 | Non-severe sepsis | | .90 |  | 1.49 | 6.50 |  |
| 23 | Non-severe sepsis | | 1.66 |  | 1.96 | 2.33 |  |
| 24 | Non-severe sepsis | | 3.19 | 2.48 | 1.67 | 1.72 | 2.81 |
| 25 | Non-severe sepsis | | 2.15 |  | 2.70 | 3.83 |  |
| 26 | Non-severe sepsis | | 1.23 |  | 2.71 | 3.18 |  |
| 27 | Non-severe sepsis | | 1.56 |  | 2.98 | 2.80 |  |
| 28 | Non-severe sepsis | | .78 |  | .73 | 2.80 |  |
| 29 | Non-severe sepsis | | 3.71 | 3.18 | 3.51 | 4.30 | 2.19 |
| 30 | Non-severe sepsis | | 2.13 | 2.13 | 2.06 | 3.24 |  |
| 31 | Non-severe sepsis | | 3.07 |  | 2.62 | 3.28 |  |
| 32 | Non-severe sepsis | | .48 | 1.25 | 1.47 | 2.34 | 2.78 |
| 33 | Non-severe sepsis | | 4.21 |  | 3.21 | 4.06 |  |
| 34 | Non-severe sepsis | | 2.19 |  | 2.33 | 2.73 |  |
| 35 | Non-severe sepsis | | .70 |  | 2.17 | 2.72 |  |
| 36 | Non-severe sepsis | | 1.25 | 1.31 | 5.45 | 3.90 | 4.13 |
| 37 | Non-severe sepsis | | 4.27 |  | 3.58 | 6.04 |  |
| 38 | Non-severe sepsis | | 3.19 |  | 5.56 | 4.48 |  |
| 39 | Non-severe sepsis | | 3.14 | 3.57 | 3.87 | 4.40 | 4.34 |
| 40 | Non-severe sepsis | | 4.21 | 3.55 | 3.91 | 5.19 | 4.78 |
| 41 | Non-severe sepsis | | 3.06 | 2.84 | 3.04 | 3.34 | 3.57 |
| 42 | Non-severe sepsis | | .97 | 1.34 | 2.06 | 3.07 | 4.56 |
| 43 | Non-severe sepsis | | 2.87 |  | 1.55 | 3.72 |  |
| 44 | Non-severe sepsis | | 2.27 |  | 2.31 | 2.60 |  |
| 45 | Non-severe sepsis | | 1.96 | 2.23 | 2.93 | 3.34 | 3.62 |
| 46 | Non-severe sepsis | | 4.83 | 3.42 | 2.73 | 3.63 | 3.04 |
| 47 | Non-severe sepsis | | 2.16 | 4.14 | 4.24 | 4.79 | 5.16 |
| 48 | Non-severe sepsis | | 2.91 | 4.38 | 2.96 | 3.70 | 2.12 |
| 49 | Non-severe sepsis | | 4.40 |  | 3.04 | 4.25 |  |
| 50 | Non-severe sepsis | | 12.14 | 8.93 | 4.50 | 4.00 | 3.33 |
| 51 | Non-severe sepsis | | 8.20 |  | 2.98 | 3.19 |  |
| 52 | Non-severe sepsis | | 10.20 | 3.08 | 3.38 | 5.40 | 4.62 |
| 53 | Non-severe sepsis | | 4.27 |  | 2.48 | 3.11 |  |
| 54 | Non-severe sepsis | | 7.05 | 4.05 | 3.34 | 4.60 | 4.50 |
| 55 | Non-severe sepsis | | 5.16 |  | 1.23 | 2.31 |  |
| 56 | Non-severe sepsis | | 10.01 | 9.22 | 3.33 | 4.43 | 3.62 |
| 57 | Non-severe sepsis | | 4.93 | 4.78 | 4.86 | 8.36 | 6.73 |
| 58 | Non-severe sepsis | | 4.30 | 3.90 | 4.16 | 7.02 | 9.52 |
| 59 | Non-severe sepsis | | 6.39 |  | 3.51 | 3.22 |  |
| 60 | Non-severe sepsis | | 10.13 | 3.38 | 3.67 | 6.31 |  |

^a^ Determined in cases with severe sepsis/septic shock.

^b^ Empty fields indicate missing data and no performed analysis.
