# Supplementary material for: Quantitative Real-Time Polymerase Chain Reaction Measurement of HLA-DRA Gene Expression in Whole Blood Is Highly Reproducible and Shows Changes That Reflect Dynamic Shifts in Monocyte Surface HLA-DR Expression during the Course of Sepsis
Source: PLoS One. 2016 May 4;11(5):e0154690. doi: 10.1371/journal.pone.0154690 (PMC4856385; doi:10.1371/journal.pone.0154690)
Supplement: S3 Table — (DOCX) [file pone.0154690.s003.docx]

**S3 Table. *CIITA* mRNA ratio (CIITA/PPIB-reference) expression in bacteraemic infection categorized by sepsis severity.**

| **ID** | **Severity** | **SOFA-**  **score** ^a^ | **CIITA**  **Day 1-2** | **CIITA**  **Day 3** ^b^ | **CIITA**  **Day 7** | **CIITA**  **Day 14** | **CIITA**  **Day 28** ^b^ |
| --- | --- | --- | --- | --- | --- | --- | --- |
| 1 | Septic shock | 6 | .01 |  | .04 | .15 |  |
| 2 | Septic shock | 5 | .05 | .03 | .05 | .24 | .38 |
| 3 | Severe sepsis | 7 | .07 |  | .09 | .24 |  |
| 4 | Severe sepsis | 7 | .03 |  | .15 | .26 | .24 |
| 5 | Severe sepsis | 7 | .11 | .12 | .20 | .32 |  |
| 6 | Severe sepsis | 6 | .14 | .14 | .21 | .25 | .39 |
| 7 | Severe sepsis | 5 | .06 | .04 | .06 | .13 | .10 |
| 8 | Severe sepsis | 5 | .08 | .09 | .12 | .22 | .12 |
| 9 | Severe sepsis | 5 | 1.15 |  | .12 | .07 | .15 |
| 10 | Severe sepsis | 4 | .07 |  | .23 | .14 |  |
| 11 | Severe sepsis | 4 | .09 | .06 | .17 | .25 | .18 |
| 12 | Severe sepsis | 3 | .12 |  | .03 | .24 |  |
| 13 | Severe sepsis | 3 | .11 | .12 | .58 | .19 |  |
| 14 | Severe sepsis | 2 | .10 |  | .18 | .22 |  |
| 15 | Severe sepsis | 2 | .03 |  | .09 | .12 |  |
| 16 | Severe sepsis | 2 | .16 | .17 | .14 | .20 | .34 |
| 17 | Severe sepsis | 1 | .11 | .13 | .20 | .38 | .48 |
| 18 | Severe sepsis | 1 | .09 | .13 | .13 | .24 | .16 |
| 19 | Severe sepsis | 1 | .08 | .12 | .12 | .17 | .20 |
| 20 | Severe sepsis | 1 | .15 |  | .15 | .16 |  |
| 21 | Non-severe sepsis | | .11 | .81 | .17 | .11 | .12 |
| 22 | Non-severe sepsis | | .06 |  | .10 | .20 |  |
| 23 | Non-severe sepsis | | .13 |  | .19 | .32 |  |
| 24 | Non-severe sepsis | | .26 | .31 | .15 | .45 | .37 |
| 25 | Non-severe sepsis | | .18 |  | .24 | .24 |  |
| 26 | Non-severe sepsis | | .93 |  | .24 | .34 |  |
| 27 | Non-severe sepsis | | .19 |  | .29 | .42 |  |
| 28 | Non-severe sepsis | | .20 |  | .12 | .49 |  |
| 29 | Non-severe sepsis | | .17 | .15 | .19 | .22 | .19 |
| 30 | Non-severe sepsis | | .32 | .24 | .25 | .40 |  |
| 31 | Non-severe sepsis | | .07 |  | .09 | .14 |  |
| 32 | Non-severe sepsis | | .04 | .07 | .10 | .19 | .24 |
| 33 | Non-severe sepsis | | .16 |  | .15 | .21 |  |
| 34 | Non-severe sepsis | | .13 |  | .12 | .37 |  |
| 35 | Non-severe sepsis | | .06 |  | .26 | .33 |  |
| 36 | Non-severe sepsis | | .09 | .07 | .19 | .23 | .29 |
| 37 | Non-severe sepsis | | .58 |  | .35 | .87 |  |
| 38 | Non-severe sepsis | | .11 |  | .24 | .26 |  |
| 39 | Non-severe sepsis | | .63 | .64 | .86 | .72 | .69 |
| 40 | Non-severe sepsis | | .40 | .52 | .73 | .58 | .79 |
| 41 | Non-severe sepsis | | .17 | .20 | .22 | .24 | .26 |
| 42 | Non-severe sepsis | | .06 | .14 | .18 | .20 | .23 |
| 43 | Non-severe sepsis | | .14 |  | .17 | .23 |  |
| 44 | Non-severe sepsis | | .08 |  | .20 | .21 |  |
| 45 | Non-severe sepsis | | .12 | .18 | .26 | .25 | .30 |
| 46 | Non-severe sepsis | | .18 | .15 | .21 | .32 | .34 |
| 47 | Non-severe sepsis | | .22 | .44 | .23 | .50 | .51 |
| 48 | Non-severe sepsis | | .12 | .12 | .25 | .30 | .15 |
| 49 | Non-severe sepsis | | .22 |  | .23 | .42 |  |
| 50 | Non-severe sepsis | | .28 | .21 | .21 | .24 | .14 |
| 51 | Non-severe sepsis | | .28 |  | .26 | .38 |  |
| 52 | Non-severe sepsis | | .20 | .14 | .18 | .37 | .33 |
| 53 | Non-severe sepsis | | .15 |  | .22 | .25 |  |
| 54 | Non-severe sepsis | | .30 | .20 | .29 | .27 | .21 |
| 55 | Non-severe sepsis | | .39 |  | .20 | .39 |  |
| 56 | Non-severe sepsis | | .41 | .49 | .47 | .76 | .46 |
| 57 | Non-severe sepsis | | .18 | .21 | .31 | .36 | .37 |
| 58 | Non-severe sepsis | | .18 | .27 | .26 | .31 | .45 |
| 59 | Non-severe sepsis | | .11 |  | .25 | .24 |  |
| 60 | Non-severe sepsis | | .39 | .17 | .24 | .23 |  |

^a^ Determined in cases with severe sepsis/septic shock.

^b^ Empty fields indicate missing data and no performed analysis.
